# Adipose-derived stromal/stem cells improve epidermal homeostasis

**DOI:** 10.1038/s41598-019-54797-5

**Published:** 2019-12-04

**Authors:** Mariko Moriyama, Shunya Sahara, Kaori Zaiki, Ayumi Ueno, Koichi Nakaoji, Kazuhiko Hamada, Toshiyuki Ozawa, Daisuke Tsuruta, Takao Hayakawa, Hiroyuki Moriyama

**Affiliations:** 1 Pharmaceutical Research and Technology Institute, Kindai University, Higashi-Osaka, Osaka 577-8502 Japan; 2 Research and Development Division, PIAS Corporation, Kobe, Hyogo 651-2241 Japan; 3 Department of Dermatology, Graduate School of Medicine, Osaka City University, Abeno-Ku, Osaka 545-8585 Japan

**Keywords:** Cell biology, Mesenchymal stem cells

## Abstract

Wound healing is regulated by complex interactions between the keratinocytes and other cell types including fibroblasts. Recently, adipose-derived mesenchymal stromal/stem cells (ASCs) have been reported to influence wound healing positively via paracrine involvement. However, their roles in keratinocytes are still obscure. Therefore, investigation of the precise effects of ASCs on keratinocytes in an *in vitro* culture system is required. Our recent data indicate that the epidermal equivalents became thicker on a collagen vitrigel membrane co-cultured with human ASCs (hASCs). Co-culturing the human primary epidermal keratinocytes (HPEK) with hASCs on a collagen vitrigel membrane enhanced their abilities for cell proliferation and adhesion to the membrane but suppressed their differentiation suggesting that hASCs could maintain the undifferentiated status of HPEK. Contrarily, the effects of co-culture using polyethylene terephthalate or polycarbonate membranes for HPEK were completely opposite. These differences may depend on the protein permeability and/or structure of the membrane. Taken together, our data demonstrate that hASCs could be used as a substitute for fibroblasts in skin wound repair, aesthetic medicine, or tissue engineering. It is also important to note that a co-culture system using the collagen vitrigel membrane allows better understanding of the interactions between the keratinocytes and ASCs.

## Introduction

Mesenchymal stem/stromal cells (MSCs) have been reported to express various cytokines and growth factors that can regenerate tissue damage^1,2^. Among these, the human adipose-derived mesenchymal stromal cells (hASCs) are able to be expanded in culture for a long period of time, and can overcome ethical concerns because they can be easily and safely obtained from autologous lipoaspirates. and grown *ex vivo* under appropriate culture conditions. Thus, hASCs are regarded as an attractive source of stem cells for cell-based therapies in regenerative medicine, aesthetic medicine, and tissue engineering.

Recently, the use of ASCs in skin wound healing has attracted great attention. Several researchers have already reported that transplanted ASCs can activate the regeneration processes in an animal-based model^3–5^. During wound healing, ASCs may influence many cell types such the immune cells, endothelial cells and fibroblasts involved in inflammation, neovascularization and scar formation, respectively^6^. Keratinocytes associated with re-epithelization can also be a target of the ASCs. Sheng *et al*. have demonstrated that transplanted ASCs enhanced the proliferation of epidermal cells, which resulted in the development of a thicker epidermis during cutaneous wound healing^5^. It has also been reported that, in *in vitro* culture systems, conditioned medium obtained using ASCs promoted the proliferation and migration of immortalized human keratinocytes^7^. These results demonstrated that ASCs can improve keratinocyte function in wound healing via paracrine involvement. Additionally, recent evidence has suggested that ASCs could modulate epidermal morphogenesis and present an advantageous, autologous cell source for skin tissue engineering^8^. Therefore, it is important to investigate the precise effects of ASCs on keratinocytes in an *in vitro* culture system.

There are mainly two types of co-culture systems depending on the state of the cells in adhesion; direct or indirect systems. In direct co-culture systems, distinct types of cells are cultured together in the same culture environment, thus allowing direct cell-cell contact with each other. In indirect co-culture systems, conditioned medium obtained from cells are used for culture the other cells, or a porous physical barrier such as a transwell membrane is used for the separation of several types of cells^9^. Transwell co-culture systems have the advantage over the direct co-culture and conditioned medium techniques in that cellular polarity is preserved. However, low porosity of commercial track-etched membranes, typically made from polyethylene terephthalate (PET) or polycarbonate (PC), limits the degree of cell-cell contact or membrane permeability. To overcome this limitation, Takezawa *et al*. have developed the “collagen vitrigel” membrane that is a thin and transparent with excellent gel strength and protein permeability^10,11^. Moreover, the properties of the high-density collagen fibrils of a collagen vitrigel membrane are equivalent to those of the connective tissues *in vivo* and provide conditions better suited for cell growth.

In this study, we have investigated the effect of hASCs on keratinocytes using a transwell co-culture system with collagen vitrigel, PET, or PC membranes. Our present work revealed that hASCs as well as human dermal fibroblasts (HNDF) have a positive impact on the keratinocytes with respect to their proliferation, stemness maintenance, and adhesiveness to membranes via paracrine involvement when co-cultured using the collagen vitrigel membrane, but demonstrate opposite effects upon use of PET or PC membranes.

## Results

### Characterization of hASCs and HNDF

**Figure 1:**
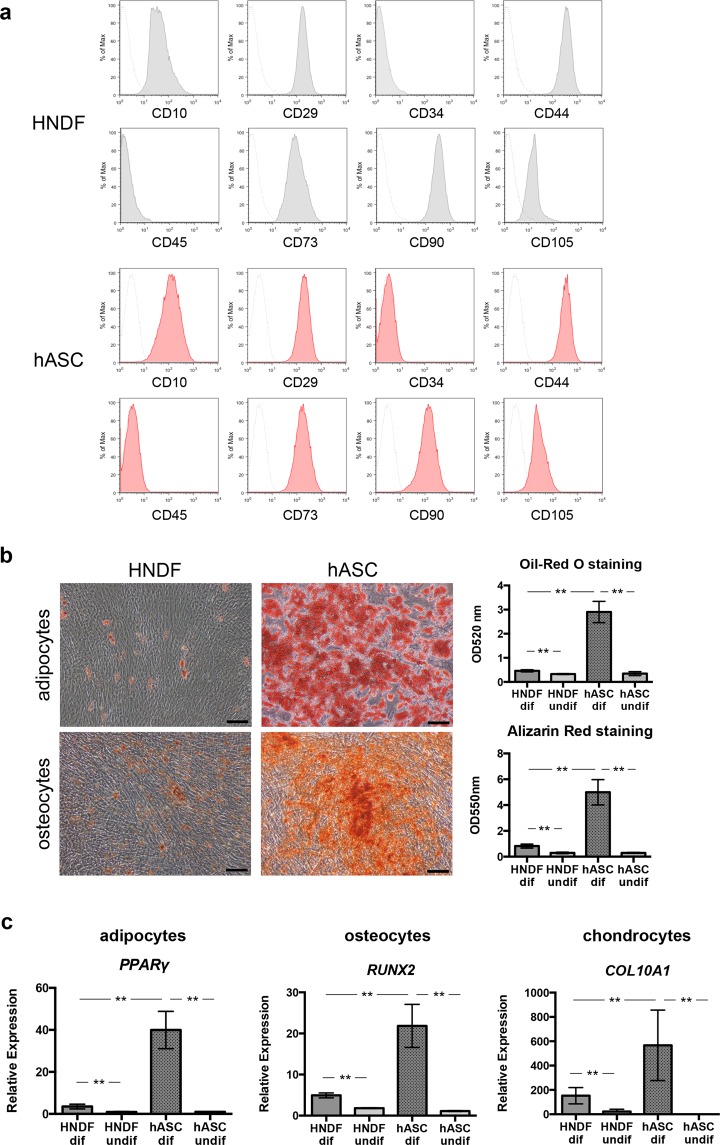
Characterization of HNDF and hASCs. (**a)** Flow cytometry analysis of HNDF and hASCs. Representative histograms are shown. **(b,c)** Differentiation potential of HNDF and hASCs for their conversion to adipocytes, osteocytes, and chondrocytes. **(b)** Representative images of Oil-Red O staining for adipocytes and Alizarin Red staining for osteocytes. The stained dye was extracted, and OD values were measured and plotted as the means of three independent experiments ± SD. Scale bars: 200 µm. **(c)** Differentiation potential into adipocytes, osteocytes, and chondrocytes were evaluated by qPCR analaysis. The graphs indicate the mean ± SE values from 3 independent experiments. **P < 0.01.

Firstly, we examined the cellular properties of hASCs and HNDF. Initially, cell surface antigens expressed on the hASCs and HNDF were analyzed by flow cytometry and no significant differences between their expression profiles were observed; the cells were consistently positive for CD10, CD29, CD44, CD73, CD90, and CD105, but negative for CD34 and CD45 (Fig. 1a). These data were consistent with previous reports describing the expression profiles of the cell surface markers of hASCs and HNDF^12–15^. Additionally, their potential for differentiation into adipocyte and osteocyte lineages was analyzed. Both hASCs and HNDF could be differentiated into adipocytes, osteocytes, and chondrocytes, but the differentiation potential of hASCs was much higher than that of the HNDF (Fig. 1b,c).

### The quality of epidermal equivalents in co-culture systems

**Figure 2:**
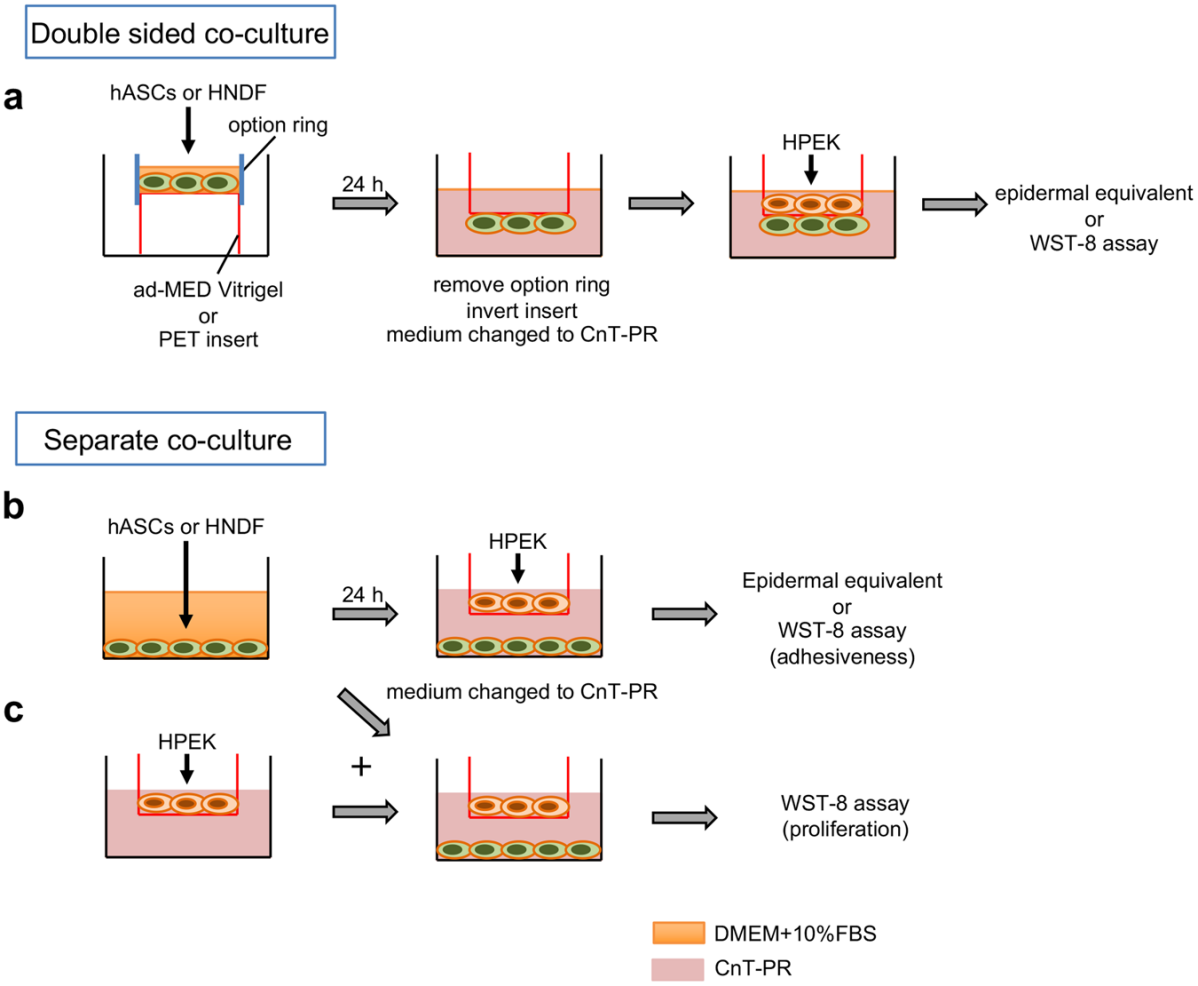
Schematic illustration of the co-culture system. (**a)** In the double-sided co-culture system, hASCs or HNDF were seeded to the back of the ad-MED Vitrigel 2 insert using Option Ring and the HPEK were then seeded to the opposite side of the insert. **(b,c)** In , either hASCs or HNDF were seeded onto 12-well culture plates. **(b)** For the adhesiveness assay, HEPK were seeded on culture inserts 24 h post seeding with hASCs/HNDF. **(c)** For the reconstitution of an epidermal equivalent, proliferation assay and qPCR analysis, HPEK were seeded in culture inserts and incubated for 24 h. The inserts were then placed in 12-well culture plates in which hASCs/HNDF were seeded.

**Figure 3:**
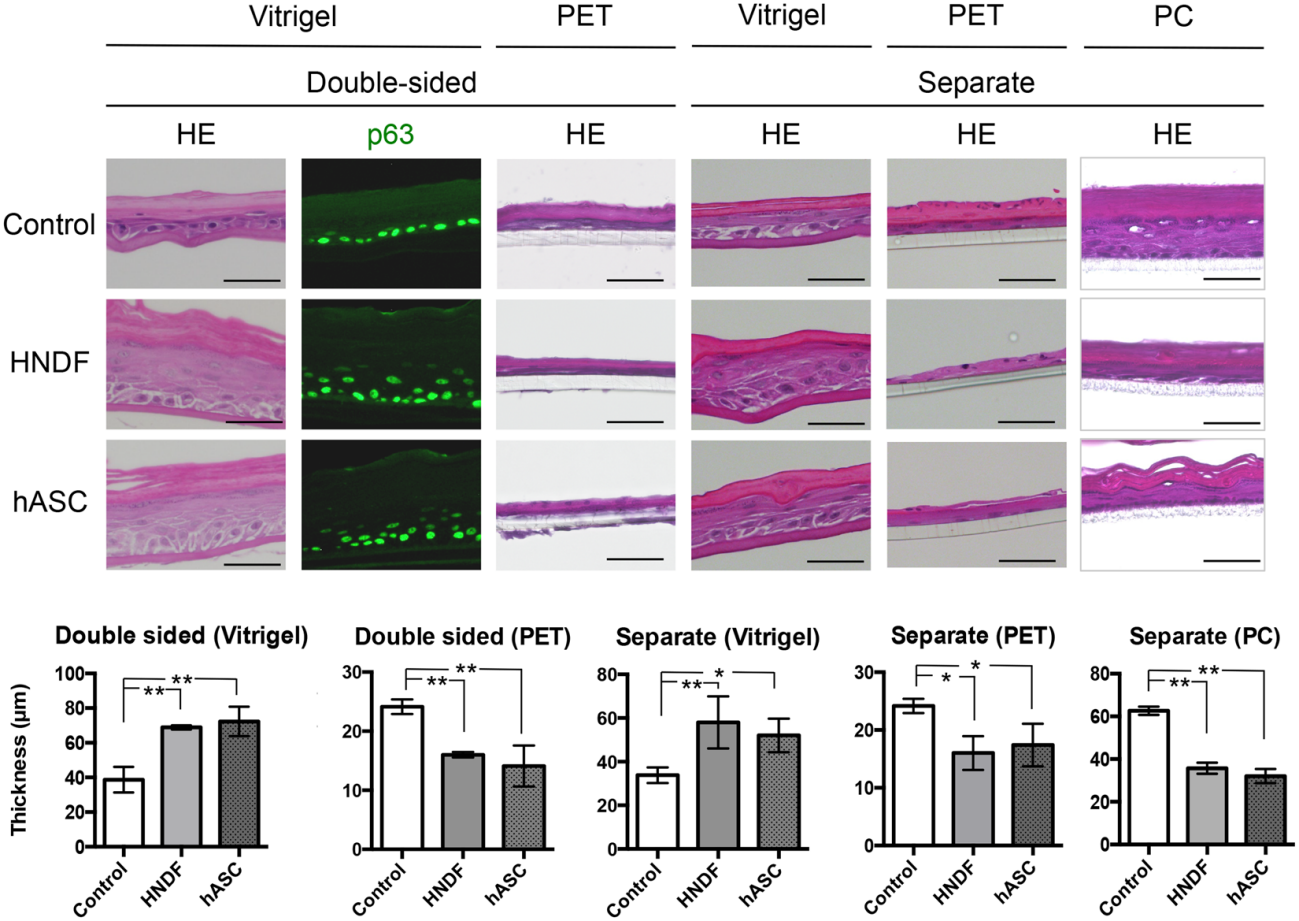
Reconstituted epidermal equivalents in a co-culture system. Epidermal equivalents were reconstituted on collagen vitrigel, PET, and PC inserts. HPEK were co-cultured with either HNDF or hASCs in double-sided or separate co-culture systems for 14 days. Epidermal equivalent reconstituted from HPEK alone (without co-culture) was defined as a control. Epidermal equivalents were then stained with hematoxylin and eosin (HE) or immunostained against p63 (green). The graphs represent the mean ± SD values for thickness of the whole epidermis of the skin equivalent in micrometers from 3–4 independent experiments. **P < 0.01, *P < 0.05. Scale bars: 50 µm.

In order to evaluate the effects of co-culture using HNDF or hASCs on epidermal development, skin epidermal equivalents were generated on collagen vitrigel, PET and PC membranes (Fig. 2a,b) and no significant difference between their qualities was observed (Fig. 3). However, the influence of co-cultures involving HNDF and hASCs on epidermal equivalents was completely opposite when inserts of the collagen vitrigel membrane and PET or PC were used. The epidermal equivalents were thicker on the collagen vitrigel membrane co-cultured with either HNDF or hASCs, either in a double-sided or separate co-culture system (Fig. 3). On the other hand, thinner epidermal equivalents were observed on PET or PC inserts co-cultured with either HNDF or hASCs (Fig. 3). We also performed an immunofluorescence staining of the epidermal equivalents against p63, a marker of the basal proliferative layer of the epidermis. The number of cells positive for p63 increased in the epidermal equivalents on the collagen vitrigel membrane co-cultured with either HNDF or hASCs in a double-sided system (Fig. 3). From these data, it can be elucidated that HNDFs and hASCs could have positive influence on HPEK in a co-culture system with the vitrigel membrane, but a negative influence when co-cultured using PET or PC inserts.

### The proliferation of keratinocytes in co-culture systems

**Figure 4:**
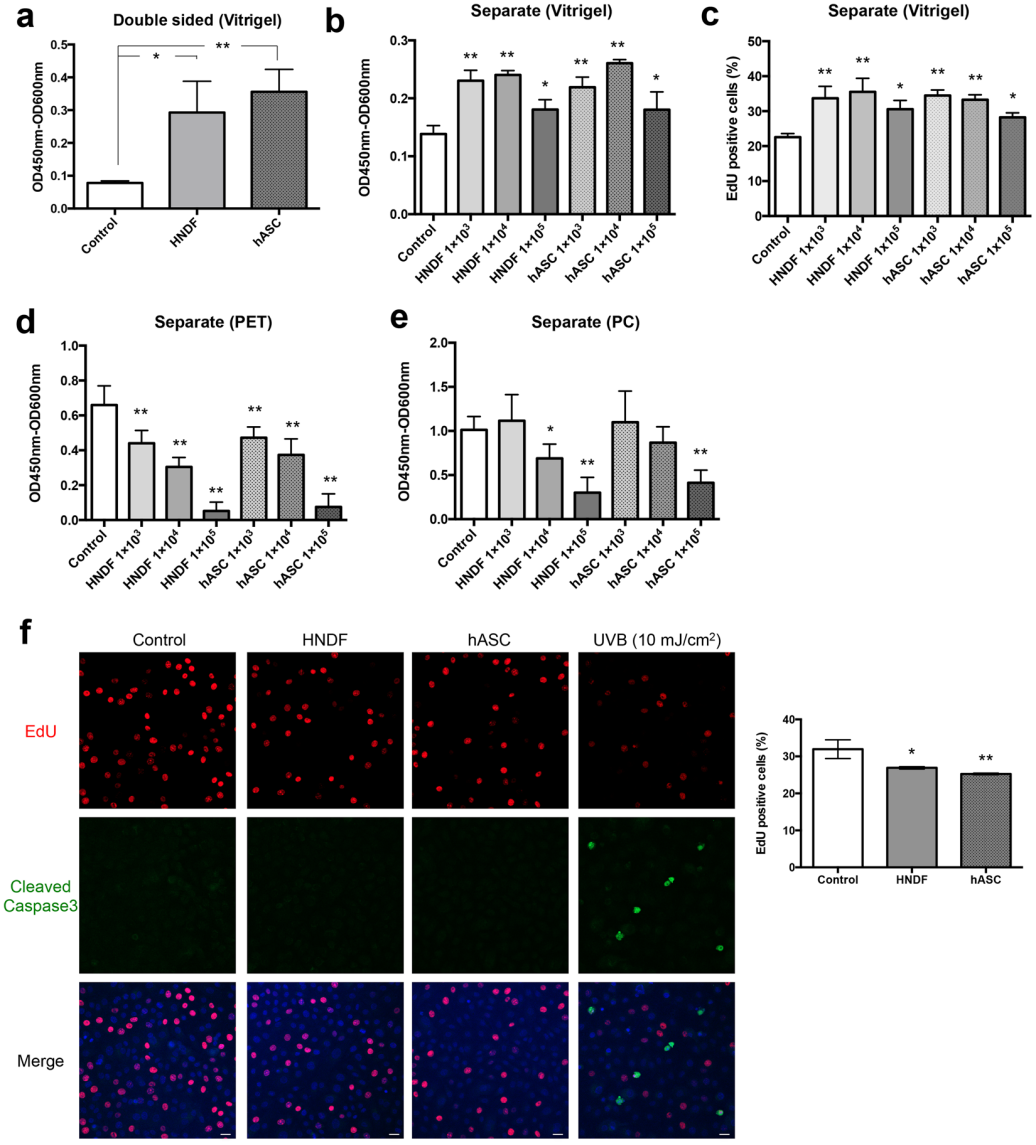
Proliferation of HPEK in co-culture system. HPEK were cultured on a vitrigel membrane insert **(a–c)**, PET insert **(d**,**f)**, and PC insert **(e)** in a double-sided co-culture system **(a**,**d)**, and separate co-culture system **(b**,**c**,**e**,**f)**. HPEK cultured alone (without co-culture) was defined as a control. Cell proliferation was evaluated by the WST-8 assay **(a**,**b**,**d**,**e)** and EdU incorporation assay **(c)**. **(a–e)** The graphs represent the mean ± SE values from 5 independent experiments. **(f)** EdU incorporation assay (red) together with immunofluorescent analysis of cleaved caspase 3 (green) were shown. Blue signal indicates nuclear staining (DAPI). The graphs indicate the mean ± SE values from 3 independent experiments. Scale bars; 20 µm. **P < 0.01, *P < 0.05.

Cell survival and proliferation are critical factors in epidermal development. Therefore, we performed a WST-8 assay to evaluate the effects of HNDF and hASCs on the proliferation of HPEK (Fig. 2a,c). The WST-8 assay revealed that the proliferation of HPEK had increased when co-cultured with hASCs or HNDF using collagen vitrigel inserts (Fig. 4a,b), which was also confirmed by conducting an EdU incorporation assay (Fig. 4c). These data indicate that either HNDF or hASCs could enhance the proliferation of HPEK in a collagen vitrigel co-culture system. Contrarily, and consistent with the results of 3D culture (Fig. 3), proliferation of HPEK had decreased significantly when co-cultured with HNDF or hASCs using PET or PC inserts in a cell-number dependent manner (Fig. 4d–f). Cell cycles of HNDF or hASCs co-cultured with HPEK were unaffected (Supplemental Fig. 1).

### Maintenance of an undifferentiated status of keratinocytes in co-culture systems

**Figure 5:**
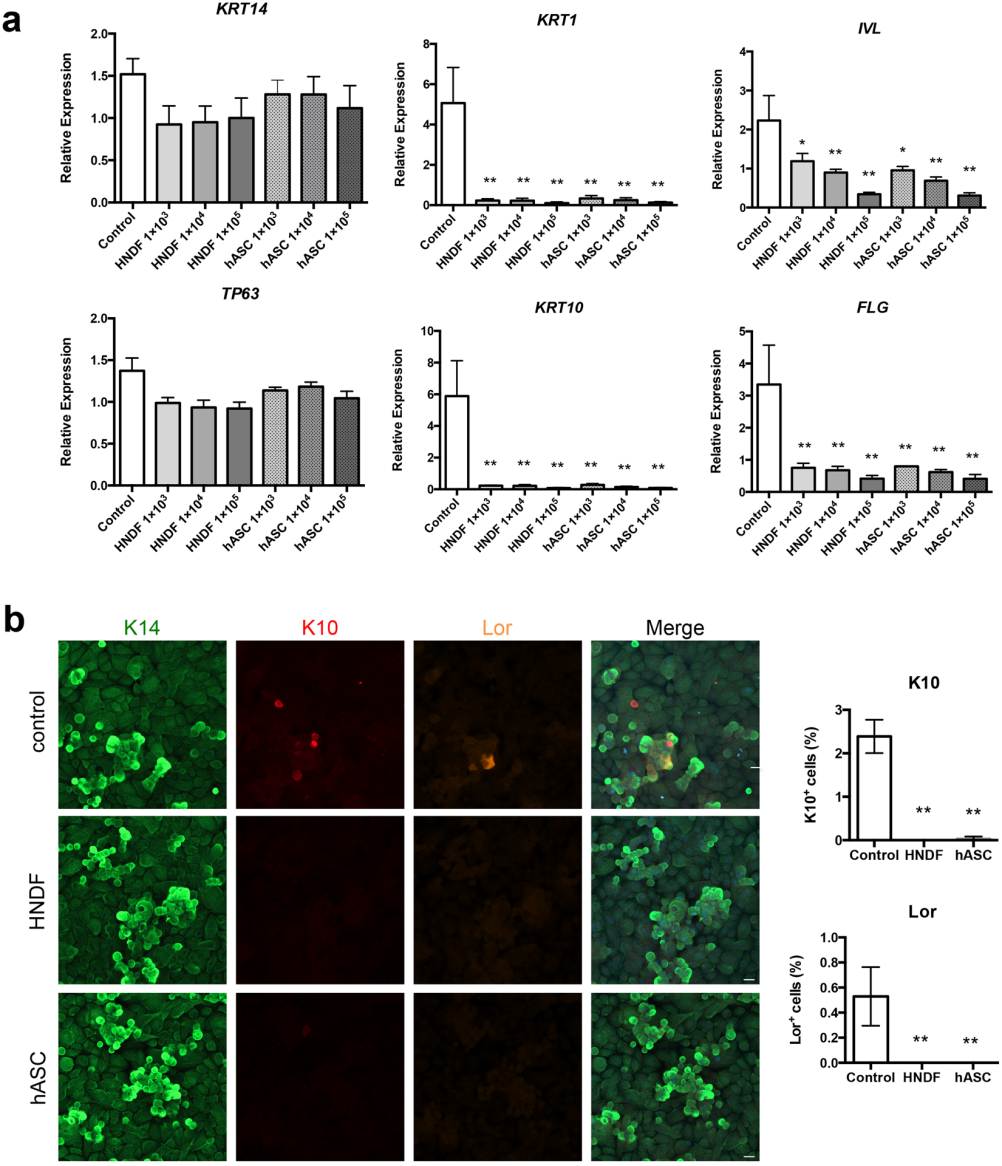
Maintenance of the undifferentiated status of HPEK in the vitrigel co-culture system. HPEK (1 × 10^5^ cells) were seeded and were cultured on the vitrigel membrane in a separate co-culture system for 24 h. HPEK cultured alone (without co-culture) was defined as a control. **(a)** Gene expression of HPEK was evaluated by qPCR analysis. The graphs represent the mean ± SE values from 3 independent experiments. **P < 0.01, *P < 0.05. **(b)** Immunofluorescent analysis of keratin 14 (K14; green), keratin 10 (K10; red), and loricrin (Lor; orange) were shown. The graphs indicate the mean ± SE values from 3 independent experiments. **P < 0.01. Scale bars; 20 µm.

We next evaluated the effect of the co-culture system on the expression of the differentiation marker of HPEK because the rate of proliferation usually decreases as cells differentiate. As expected, the expressions of the basal cell markers *KRT14* and *TP63* (deltaNp63) were unchanged whereas differentiation markers of keratinocytes were significantly downregulated at both mRNA and protein levels when co-cultured with HNDF or hASCs in collagen vitrigel inserts as we expected (Fig. 5a,b).

### The adhesive property of keratinocytes in co-culture

**Figure 6:**
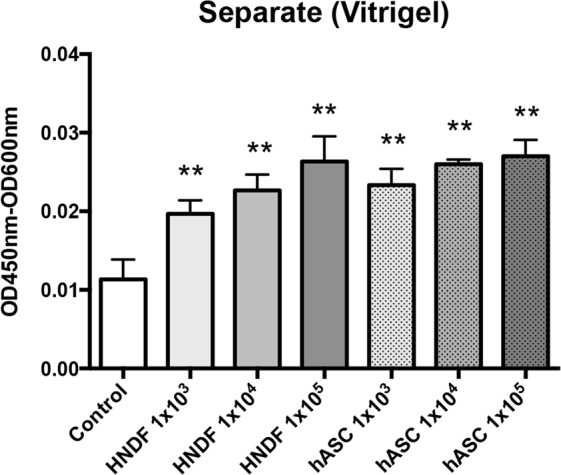
The adhesive property of HPEK in a co-culture system. HPEK were cultured on the vitrigel membrane in a separate co-culture system for 24 h. HPEK cultured alone (without co-culture) was defined as a control. Cell viability of HPEK was evaluated by the WST-8 assay. The graphs represent the mean ± SE values from 5 independent experiments. **P < 0.01.

Finally, we evaluated the effect of the co-culture system on the adhesion of cells to the collagen vitrigel membrane. Collagen vitrigel inserts were placed on 12-well plates seeded with HNDF or hASCs. These were then seeded with HPEK and cultured for 24 h to allow their attachment to the membrane (Fig. 2b). As shown in Fig. 6, the adhesive property of HPEK to collagen vitrigel inserts was improved when co-cultured with HNDF or hASCs.

## Discussion

The interaction between fibroblasts and keratinocytes and their roles in wound healing have previously been well-studied^16^. Recent studies suggest that MSCs also impact wound healing positively via a paracrine mechanism^3–5^. However, the use of ASCs in wound healing applications is still limited compared to that of the bone marrow-derived MSCs because very few basic studies have been conducted on them. Moreover, the effects of ASCs on keratinocytes are largely unknown.

Although the molecular characteristics of MSCs, including ASCs, have been extensively studied, it is still difficult to distinguish MSCs from other stromal cells such as the fibroblasts based on their surface proteins. The Mesenchymal and Tissue Stem Cell Committee of the International Society for Cellular Therapy has defined that all MSCs express CD105, CD73, and CD90 and lack the expression of either CD45, CD34, CD14 or CD11b, CD79a or CD19, and HLA-DR^15^. Other surface antigens generally expressed by the MSCs include CD10, CD29, and CD44^13,17^. However, majority of these surface markers are also expressed by fibroblasts. Consistent with this phenomenon, we also confirmed that both HNDF and ASCs were positive for CD10, CD29, CD44, CD73, CD90, and CD105, but negative for CD34 and CD45 (Fig. 1a). Some researchers have identified genes that are differentially expressed in HNDF and MSCs^13,18^. However, due to a diversity in the expression profiles of surface molecules found among MSCs isolated from different sources, there is still no consensus on the exact markers that can distinguish MSCs from HNDF.

Another important characteristic of MSCs is their potential to differentiate into fat, bone, and cartilage cells. However, clonal analysis has revealed that dermal fibroblasts are a heterogeneous cell population comprising progenitors with varying abilities to differentiate into adipogenic, osteogenic, and chondrogenic lineages^19,20^. In our study, it was observed that HNDF could differentiate into adipocytes and osteocytes when cultured in certain differentiation media, but their differentiation potential was much lower than that of the hASCs (Fig. 1b). Based on the report from Chen *et al*.^19^, 20–30% of clones from dermal fibroblasts could differentiate into adipocytes and/or osteocytes. Thus, our data are consistent with the previous results. Recently, multipotent progenitor cells known as “dermal MSCs”^21^ or “skin-derived precursors (SKPs)”^22^ were isolated from the dermis, though it is still unclear whether these populations exist in cultured fibroblasts. Additionally, recent studies have suggested that, similar to MSCs, fibroblasts also possess anti-inflammatory, immune modulatory and regenerative properties. Therefore, based on currently accepted definitions for cultured MSCs and fibroblasts, no definite properties that could characteristically distinguish between them might be known^23,24^. In our current study, we also found that both ASCs and HNDF have a positive impact on proliferation, stemness maintenance, and adhesiveness to the collagen membrane of keratinocytes (Figs. 3–6). Therefore, ASCs as well as dermal fibroblasts might prove to be attractive sources of regenerative, aesthetic, and anti-aging medicines for the epidermis.

Fibroblasts enhance the cultivation of keratinocytes, and, upon treatment with mitomycin C, they have been used extensively as feeder layers for human keratinocytes *in vitro*. Previous data demonstrated that conditioned medium from fibroblast could not promote keratinocyte proliferation^25,26^. A recent report by Wang *et al*. also revealed that the keratinocytes in direct contact with fibroblasts could proliferate and migrate faster than those separated from each other due to the presence of a transwell insert^27^. Therefore, direct cell-to-cell contact of keratinocytes with fibroblasts may be important for enhanced keratinocyte proliferation. However, under *in vivo* conditions, the keratinocytes are not in direct contact with the fibroblasts. Moreover, in our co-culture system, both double-sided and separate co-culturing with ASCs or fibroblasts using the collagen vitrigel membrane, but not the PC or PET membranes, could improve the keratinocyte activity (Figs. 3–6). The co-culture system using transwell inserts with a PC or PET membrane is a simple way to assess the changes mediated by paracrine factors in the absence of cell-cell contact. This model is reproducible and has the ability to identify the effects of co-culture on individual populations, although extensive cell growth could cover the pores of the inserts, which may result in an insignificant co-culture response. Contrarily, the collagen vitrigel membrane is a thin and transparent membrane with excellent protein permeability^11^, which allows several types of cells to be physically separated and co-cultured without direct cell-to-cell contact^28^. Takezawa *et al*. have reported that >100 kDa proteins could pass through the collagen vitrigel membrane^29^. Additionally, properties of the high-density collagen fibrils of this membrane are equivalent to those of the connective tissues *in vivo* and provide more suitable conditions for cell growth. Therefore, it is possible that co-culture with collagen vitrigel membranes may reflect the influence of fibroblasts on keratinocytes more accurately than that of the PC or PET membranes. In this study, however, we could not explain why the growth of keratinocytes co-cultured with ASCs or fibroblasts using PC or PET membranes was slower than that of the control keratinocytes. Further analysis will be required to resolve this.

In conclusion, owing to the positive effect of hASCs on epidermal keratinocytes, they can be used as a substitute for fibroblasts in skin wound repair, aesthetic medicine, or anti-aging medicine. In addition, co-culture systems using collagen vitrigel membranes enables us to analyze the individual cells separately, thus allow a precise understanding of the interactions between the keratinocytes and ASCs/fibroblasts.

## Methods

### Adipose tissue samples

Subcutaneous adipose tissue samples (10–50 g each) were taken from discarded tissue resected during plastic surgery. The study protocol was approved by the Review Board for Human Research of Osaka City University Graduate School of Medicine, and by the Kindai University Pharmaceutical Research and Technology Institute (reference number: 12-043). Each subject provided signed informed consent, and all procedures were performed in accordance with the relevant local and national guidelines and regulations.

### Cell culture

The hASCs were isolated as previously reported^2,17,30^, and maintained in alpha modified Eagle’s medium (α-MEM) comprising 10% fetal bovine serum, and 10 ng/mL epidermal growth factor (PeproTech, Rocky Hill, NJ, USA). The cells were plated on fibronectin-coated dishes at a seeding density of 4 × 10^3^ cells/cm^2^. The medium was replaced every 2 days. To obtain a hypoxic culture system, the cells were cultured in a gas mixture comprising 90% N_2_, 5% CO_2_, and 5% O_2_. A ProOx C21 carbon dioxide and oxygen controller and a C-Chamber (Biospherix, Redfield, NY, USA) were used to maintain the hypoxic conditions using the gas mixture. ASCs at passages 2–4 were used for the experiment. HNDF were purchased from Lonza (Basal, Switzerland) and maintained in Dulbecco’s modified Eagle’s medium (DMEM) containing 10% fetal bovine serum. HNDF at passages 3–5 were used for the experiment. The cells were plated at a seeding density of 4 × 10^3^ cells/cm^2^. The medium was replaced every 2 days. HPEK were purchased from CELLnTEC (Bern, Switzerland) and maintained in CnT-Prime (CELLnTEC) culture medium according to the manufacturer’s protocol. The human skin equivalents were generated using CnT-Prime-3D Barrier culture medium (CELLnTEC) according to the manufacturer’s protocol.

### Flow cytometry analysis

Flow cytometry analysis was performed as described previously^30^. Briefly, cells at passage 2 were harvested and re-suspended in a staining buffer (phosphate-buffered saline (PBS) containing 1% BSA, 2 mM EDTA and 0.01% sodium azide) to obtain a density of 1 × 10^6^ cells/mL, after which, they were incubated for 20 min on ice with a phycoerythrin (PE)-conjugated antibody against CD10, CD29, CD34, CD44, CD45, CD73, CD90, and CD105 (BioLegend, San Diego, CA, USA). Non-specific staining was assessed using the relevant isotype controls. Dead cells were excluded using the Live/Dead Fixable Far-Red Dead Cell Stain Kit (Life Technologies).

### Adipogenic, osteogenic and chondrogenic differentiation procedures

To evaluate adipogenic differentiation, cells were cultured in differentiation medium (Zen-Bio, NC, USA). After 7 days, 50% of the medium was replaced with the adipocyte medium (Zen-Bio) and this was repeated every 3 days. After 3 weeks of incubation, the adipogenic differentiation was confirmed by microscopic observation of intracellular lipid droplets with the aid of the Oil Red O staining, and by qPCR analysis. Osteogenic differentiation was induced by culturing the cells in osteocyte differentiation medium (Zen-Bio). Differentiation was examined using Alizarin Red staining, and qPCR analysis. To evaluate chondrogenic differentiation, 2 × 10^5^ cells were centrifuged at 400 × *g* for 10 min. The resulting pellets were cultured in chondrogenic medium (Lonza, Basel, Switzerland) for 14 days. Then the pellets were harvested and analyzed by qPCR analysis.

### Co-culture

In the double-sided co-culture system, hASCs or HNDF (2.5 × 10^4^ cells/cm^2^) were seeded to back side of the ad-MED Vitrigel 2 (Kanto Chemical, Tokyo, Japan) or Falcon cell culture inserts for 12-well plate (0.4 µm pore size, PET, Corning, Corning, NY, USA) using the Option ring (Kanto Chemical) in 12-well culture plates. Twenty-four hours later, medium in 12-well plates was removed, washed twice with PBS, and replaced with CnT-PR medium. Then the HPEK (8 × 10^3^ cells/cm^2^) were seeded to the opposite side of the insert (Fig. 2a). In the separate co-culture system, hASCs or HNDF (the cell seeding density were indicated in Figs. 4–6) were seeded to 12-well culture plates. After 24 h, medium in 12-well plates was removed, washed twice with PBS, and replaced with CnT-PR medium. Then the HEPK (8 × 10^3^ cells/cm^2^) were seeded on culture inserts (Fig. 2b). Alternatively, HPEK were seeded in the culture inserts, incubated for 24 h and then placed in the 12-well culture plates (Fig. 2c).

### Cell viability and adhesion assay

Cell viability was assessed using the WST-8 Assay (Dojindo Laboratories, Kumamoto, Japan), performed according to the manufacturer’s instructions. In a double-sided co-culture system, the HNDF or hASCs were gently removed from their respective sides on the membranes with a cotton swab before performing the assay. The inserts were transferred to the new 12 well culture plates, and medium inside of the insert was replaced with WST-8 solution (WST-8 were diluted at 1/10 with fresh CnT-PR medium). After 1 h incubation, the orange water-soluble formazan from WST-8 were measured for absorbance at a wavelength of 450 nm using Synergy H4 (Bio Tek, Winooski, VT, USA). To evaluate the cell adhesion, assays were performed as previously described^31^. Briefly, the HEPK (8 × 10^3^ cells/cm^2^) were seeded on culture inserts, and allowed to adhere for 24 h. Nonadherent cells were removed in a washing step with PBS. Then the inserts were transferred to the new 12 well culture plates, and remaining cells were incubated with WST-8 solution (WST-8 were diluted at 1/10 with fresh CnT-PR medium) for 1 h. The resultant formazan were measured for absorbance at a wavelength of 450 nm using Synergy H4.

### EdU proliferation assay and immunofluorescent staining

Cell proliferation was detected by incorporating 5-ethynyl-2′-deoxyuridine (EdU) into the cell culture system and fluorescence imaging of the cells using a Click-iT EdU Alexa Fluor 488 or Alexa Fluor 647 Imaging Kit (Thermo Fisher Scientific) according to the manufacturer’s protocol. Briefly, the cells were incubated with 10 µM EdU for 2 h before fixation. The fixed cells were then permeabilized and stained with Alexa 488- or Alexa Fluor 647-conjugated azide. For immunofluorescent staining, the cells were incubated with PBSMT (PBS containing 2% skim milk and 0.1% Triton X-100) for 1 h, and incubated with rabbit monoclonal antibody against Cleaved Caspase 3 (Cell Signaling Technology, Danvers, MA, USA, 1:400), or chick polyclonal antibody against keratin 14 (BioLegend, 1:1000), mouse monoclonal antibody against keratin 10 (BioLegend, 1:1000), and rabbit polyclonal antibody against loricrin (BioLegend, 1:1000) overnight at 4 °C. After being washed, the cells were incubated with Alexa Fluor 488 conjugated-donkey polyclonal antibody against rabbit IgG, or Alexa Fluor 488 conjugated-donkey polyclonal antibody against chick IgY, Alexa Fluor 647 conjugated-donkey polyclonal antibody against mouse IgG, and Alexa Fluor 546 conjugated-donkey polyclonal antibody against rabbit IgG. Following the final washing step, the membranes with cells were cut away from the inserts, and mounted with coverslips by using ProLong Gold Antifade Mountant with DAPI (Thermo Fisher Scientific). Images were obtained using a fluorescence microscope (BZ-9000; Keyence, Osaka, Japan) and the number of EdU-, keratin 10-, or loricrin-positive cells were analyzed by the IN Cell Investigator Software (GE Healthcare, Buckinghamshire, UK). No fewer than 10 fields and totally 2,000 cells were counted for each sample.

### RNA extraction, complementary DNA (cDNA) generation, and quantitative polymerase chain reaction (qPCR)

Total RNA was extracted from 2 × 10^5^ cells using a PureLink RNA Mini Kit (Thermo Fisher Scientific) according to the manufacturer’s instructions. For cDNA synthesis, digestion by on-column DNase I was performed using the PureLink DNase Set (Thermo Fisher Scientific), followed by random primer-mediated reverse transcription using a M-MLV reverse transcriptase (Promega) with 1 µg of total RNA as input. The cDNA was purified using a MinElute PCR Purification Kit (Qiagen, Hilden, Germany). The qPCR analysis was performed and reported according to the Minimum Information for Publication of Quantitative Real-Time PCR Experiments (MIQE) guidelines^32^. All reactions were performed in 96-well plates using the Applied Biosys7300 real-time PCR system (Thermo Fisher Scientific). Reactions were performed in a total volume of 15 µl, containing of 7.5 µl of 2 × Power SYBR Green PCR Master Mix (Thermo Fisher Scientific), 5 ng cDNA (total RNA equivalents), and 200 nM of each primer (final concentration). The thermal cycling protocol used for PCR was as follows: initial denaturation for 10 min at 95 °C followed by 40 cycles of 15 s at 95 °C and 1 min at 60 °C. The fluorescence signal was measured at the end of each annealing/extension step at 60 °C. After the amplification step, a melting curve analysis with a temperature gradient of 0.5 °C/s from 65 °C to 95 °C was performed to confirm that the specific products only were amplified. All qPCR reactions were performed in triplicates and the Cq values were averaged. The relative expression level of each gene was calculated using the ΔΔCt method, and the most reliable reference gene was identified from the eight genes (*ACTB*, *B2M*, *GAPDH*, *GUS*, *H6PD*, *UBC*, *UBE2D2*, and *UBE4D*) using the genorm^PLUS^  module in qbase^PLUS^ software (Biogazelle, Zwijnaarde, Belgium). Details of the primers used in these experiments are provided in Table 1.

**Table 1:** Primers used in this study.

Gene		Primer sequence (5′ → 3′)
*KRT14*	F	CCTCCTCCAGCCGCCAAATCC
	R	TTGGTGCGAAGGACCTGCTCG
*TP63*	F	CTGGAAAACAATGCCCAGAC
	R	GGGTGATGGAGAGAGAGCAT
*KRT10*	F	TGATGTGAATGTGGAAATGAATGC
	R	GTAGTCAGTTCCTTGCTCTTTTCA
*KRT1*	F	ATTTCTGAGCTGAATCGTGTGATC
	R	CTTGGCATCCTTGAGGGCATT
*IVL*	F	TCCTCCAGTCAATACCCATCAG
	R	CAGCAGTCATGTGCTTTTCCT
*FLG*	F	ATGAGCAGGCACGAGACAA
	R	TGTCCACGAATGGTGTCCT
*PPARG*	F	TACTGTCGGTTTCAGAAATGCC
	R	GTCAGCGGACTCTGGATTCAG
*RUNX2*	F	CCGCCTCAGTGATTTAGGGC
	R	GGGTCTGTAATCTGACTCTGTCC
*COL10A1*	F	GGGGCTAAGGGTGAAAGGG
	R	GGTCCTCCAACTCCAGGATCA
*GAPDH*	F	GCTCTCTGCTCCTCCTGTTC
	R	ACGACCAAATCCGTTGACTC
*B2M*	F	TATCCAGCGTACTCCAAAGA
	R	GACAAGTCTGAATGCTCCAC
*UBC*	F	ATTTGGGTCGCGGTTCTTG
	R	TGCCTTGACATTCTCGATGGT
*UBE2D2*	F	TGGCAAGCTACAATAATGGGG
	R	GGAGACCACTGTGATCGTAGA

### Histology

Skin epidermal equivalents were fixed in 4% paraformaldehyde, embedded in an optimal cutting temperature compound, frozen, and sectioned into samples of 10 μm thickness. The sections were then subjected either to hematoxylin and eosin staining or immunohistochemical analysis, as described previously^33^. The sections were stained with a mouse monoclonal antibody against p63 (abcam) diluted at 1:100. After the sections were washed with 0.1% Triton X100 containing PBS, they were incubated with the Alexa 488 conjugated-donkey polyclonal antibody against rabbit IgG. Images were obtained using a fluorescence microscope (BZ-9000) and analyzed using the BZ-Analyzer Software (Keyence).

### Statistical analysis

Statistical differences were determined by one-way analysis of variance (ANOVA) followed by the Dunnett’s or Tukey’s test using a GraphPad Prism software (GraphPad Software, La Jolla, CA, USA). A value of *P* < 0.05 was considered statistically significant (**P < 0.01, *P < 0.05).

## Supplementary information

Supplementary information
