# Supplementary material for: Adipose-derived stromal/stem cells improve epidermal homeostasis
Source: Sci Rep. 2019 Dec 4;9:18371. doi: 10.1038/s41598-019-54797-5 (PMC6892794; doi:10.1038/s41598-019-54797-5)
Supplement: Supplementary file 1 — Supplementary information [file 41598_2019_54797_MOESM1_ESM.docx]

**Adipose-derived stromal/stem cells improve epidermal homeostasis**

- Mariko Moriyama^1^, Shunya Sahara^2^, Kaori Zaiki^2^, Ayumi Ueno^2^, Koichi Nakaoji^2^, Kazuhiko Hamada^2^, Toshiyuki Ozawa^3^, Daisuke Tsuruta^3^, Takao Hayakawa^1^, Hiroyuki Moriyama^1*^
- ^1^Pharmaceutical Research and Technology Institute, Kindai University, Higashi-Osaka, Osaka 577-8502, Japan
- ^2^Research and Development Division, PIAS Corporation, Kobe, Hyogo 651-2241, Japan

^3^Department of Dermatology, Graduate School of Medicine, Osaka City University, Abeno-Ku, Osaka 545-8585, Japan

- **Corresponding Author:**
- Hiroyuki Moriyama, Ph.D.
- Address: 3-4-1 Kowakae, Higashi-Osaka, Osaka 577-8502 JAPAN
- Telephone and Fax numbers: +81-6-4307-4312
- E-mail address: moriyama@phar.kindai.ac.jp

**SUPPLEMENTARY METHODS**

**Cell cycle assay**

Cells were incubated with 10 µM of 5-ethynyl-2′-deoxyuridine (EdU) for 2 h. Then cell cycle analyses were conducted using the Click-iT EdU Alexa Fluor 488 Flow Cytometry assay kit (Thermo Fisher Scientific), according to the manufacturer's instructions. FxCycle Far Red (Thermo Fisher Scientific) was used to stain cells for DNA content. Cells were analyzed by flow cytometry (ec800 cell analyzer, SONY, Tokyo, Japan). FlowJo (TreeStar Inc., Ashland, OR, USA) software was used for quantitative analysis.

**SUPPLEMENTARY FIGURE LEGENDS**

**Supplementary Figure 1. The effect of PET/vitrigel membrane on HNDF/hASCs.** HNDF and hASCs (1x10^4^ cells) were seeded on 12 well culture plate, and HPEK (1x10^4^ cells/cm^2^) were seeded on PET or vitrigel inserts. After 24 h, the inserts were moved to the 12 well culture plates where HNDF/hASCs were seeded (day 1). **(a)** Cell cycle analysis of HNDF/hASCs on day 4. (b) Representative phase images of HNDF/hASCs on day 1 and day 4. Scale bars; 200 µm.
